# Supplementary material for: ProteinShader: illustrative rendering of macromolecules
Source: BMC Struct Biol. 2009 Mar 30;9:19. doi: 10.1186/1472-6807-9-19 (PMC2672931; doi:10.1186/1472-6807-9-19)
Supplement: Additional file 1 — ProteinShader program without source code. This compressed file contains the complete ProteinShader program including associated libraries, but no source code. A README.txt file gives an overview of the ProteinShader distribution, and the index.html file in the help subdirectory has directions on getting started with the program as well as a set of tutorials. [file 1472-6807-9-19-S1.zip › ProteinShader-beta-0_9_4-binary/help/api/org/proteinshader/structure/visitor/enums/class-use/ResidueMode.html]

Uses of Class org.proteinshader.structure.visitor.enums.ResidueMode (ProteinShader API)


|  |  |  |
| --- | --- | --- |
| **Overview** \| **Package** \| **Class** \| **Use** \| **Tree** \| **Deprecated** \| **Index** \| **Help** | |  |
| PREV   NEXT | **FRAMES**    **NO FRAMES**     **All Classes** |  |


---


## **Uses of Class org.proteinshader.structure.visitor.enums.ResidueMode**

| Packages that use ResidueMode | |
| --- | --- |
| **org.proteinshader.structure** | Holds the classes that store information from a Protein Data Bank file: Structure, Model, Chain, AminoAcid, Heterogen, Water, Atom, Bond, Helix, BetaStrand, Loop, *etc*. |
| **org.proteinshader.structure.visitor** | Holds Visitor classes that know how to traverse the hierarchy of objects held by class Structure. |
| **org.proteinshader.structure.visitor.enums** | Holds enums needed by the Visitor classes. |

Uses of ResidueMode in org.proteinshader.structure

| Methods in org.proteinshader.structure with parameters of type ResidueMode | |
| --- | --- |
| `void` | `Structure.clearBonds(ResidueMode residueMode)`             Clears Bonds from the Structure. |

Uses of ResidueMode in org.proteinshader.structure.visitor

| Methods in org.proteinshader.structure.visitor with parameters of type ResidueMode | |
| --- | --- |
| `void` | `Visitor.setMode(ResidueMode residueMode)`             Sets the traversal mode with regard to what kind of Residues should be visited: ALL, AMINO\_ACIDS, AA\_AND\_HETEROGENS, AA\_AND\_WATERS, HETEROGENS, WATERS, HET\_AND\_WATERS, or NONE. |

Uses of ResidueMode in org.proteinshader.structure.visitor.enums

| Methods in org.proteinshader.structure.visitor.enums that return ResidueMode | |
| --- | --- |
| `static ResidueMode` | `ResidueMode.valueOf(String name)`             Returns the enum constant of this type with the specified name. |
| `static ResidueMode[]` | `ResidueMode.values()`             Returns an array containing the constants of this enum type, in the order they're declared. |

---


|  |  |  |
| --- | --- | --- |
| **Overview** \| **Package** \| **Class** \| **Use** \| **Tree** \| **Deprecated** \| **Index** \| **Help** | |  |
| PREV   NEXT | **FRAMES**    **NO FRAMES**     **All Classes** |  |


---

# *Copyright © 2007-2008*
